# Out with the Old and in with the New—Is Backward Inhibition a Domain-Specific Process?

**DOI:** 10.1371/journal.pone.0142613

**Published:** 2015-11-13

**Authors:** Francesca Foti, Stefano Sdoia, Deny Menghini, Stefano Vicari, Laura Petrosini, Fabio Ferlazzo

**Affiliations:** 1 Department of Psychology, University “Sapienza” of Rome, Rome, Italy; 2 IRCCS Fondazione Santa Lucia, Rome, Italy; 3 Child Neuropsychiatry Unit, Neuroscience Department, “Children’s Hospital Bambino Gesù”, Rome, Rome, Italy; Center for BrainHealth, University of Texas at Dallas, UNITED STATES

## Abstract

Effective task switching is supported by the inhibition of the just executed task, so that potential interference from previously executed tasks is adaptively counteracted. This inhibitory mechanism, named Backward Inhibition (BI), has been inferred from the finding that switching back to a recently executed task (A-B-A task sequence) is harder than switching back to a less recently executed task (C-B-A task sequence). Despite the fact that BI effects do impact performance on everyday life activities, up to now it is still not clear whether the BI represents an amodal and material-independent process or whether it interacts with the task material. To address this issue, a group of individuals with Williams syndrome (WS) characterized by specific difficulties in maintaining and processing visuo-spatial, but not verbal, information, and a mental age- and gender-matched group of typically developing (TD) children were subjected to three task-switching experiments requiring verbal or visuo-spatial material to be processed. Results showed that individuals with WS exhibited a normal BI effect during verbal task-switching, but a clear deficit during visuo-spatial task-switching. Overall, our findings demonstrating that the BI is a material-specific process have important implications for theoretical models of cognitive control and its architecture.

## Introduction

The capacity to inhibit thoughts and actions that are no longer relevant (but that were relevant right earlier) is crucial for everyday functioning. Responding to a new situation or task requires the deactivation of mindsets that have been adopted in the recent past in favor of currently relevant mindsets [1]. On the other hand, in a changing environment, the overall success also depends on the ability to complete a task while resisting the tendency to skip to another task for no other reason than its presence in the field of view [2]. Thus, flexibility is as important as stability in directing goal-related behavior. With regard to the complementary nature of these two needs, the *stability/flexibility paradox* has been introduced to describe the need to resist tendencies toward goal-incongruent actions and the need to change control settings flexibly [2].

To examine the interplay of flexibility- and stability-oriented cognitive processes, task-switching paradigms are usually implemented, in which participants perform one of two or more tasks with identical stimuli in each experimental trial, with a task cue indicating the relevant task [3,4]. Sometimes the task is changed (i.e., B-A) and sometimes it is repeated from the previous trial (i.e., A-A). Switching from one task to another implies a performance cost, as evidenced by the increase in reaction times (RTs) and error rates after a task switch compared to the subsequent performance on the same task [3,4]. This difference between switching and repeating the task is referred to as the switch cost [5,6]. Importantly, switching back to a recently executed task (such as performing the A task as the third trial in an A-B-*A* sequence of tasks) is harder than switching back to a less recently executed task (such as performing the A task as the third trial in a C-B-*A* sequence of tasks), as evidenced by the slowing of RTs on the third trial in an A-B-A sequence compared to the third trial in a C-B-A sequence [1]. Such an effect has been interpreted as due to the necessity to suppress the mental representation (task set) of the just executed task in order to reduce its potential interference on the new task [7]. The consequence of such an inhibitory mechanism named Backward Inhibition (BI; [1–4,8]) is that when a previously inhibited task is reengaged (as in A-B-*A*) it still suffers from a residual persisting inhibition. Overcoming this inhibition requires resources that cannot be allocated to the current task. Consequently, task performance is worse (i.e., increased RTs and error rates) on the third trial in the A-B-A sequence compared to the C-B-A sequence.

Hence, the BI is assumed to subserve efficient task switching, allowing individuals to be sufficiently flexible to efficiently adapt to continuously changing environments [1,9]. Poor inhibitory control of the task that was just executed could reduce cognitive flexibility and decrease the overall efficiency of the system.

Although the role of inhibition in task-switching process has received increasing attention with regard to executive control, our understanding of the factors that modulate inhibitory processes remains poor. Many studies have examined the BI effects [1–4,8], but whether and how inhibitory control is linked to the nature of the information that is to be processed have not been addressed, leaving unanswered the possibility of material-specific differences in BI-related inhibitory control. Specifically, no study has yet examined whether material-specific effects are present in the BI process, as it has been reported for working memory [10], conflict resolution [11,12], and attentional control [13,14]. While evidence exists that BI can be triggered at separated stage of information processing (e.g., during task preparation [15]; during task stimulus processing [16]; and during task response selection [17]) depending on the stage at which the task conflict is detected, whether BI also depends on the type of input material it is triggered by is still an open issue. One hypothesis is that BI operates on task representations regardless of the type of input from which task representations are generated (e.g., verbal or spatial). Some evidence exists that seems to support the idea that BI operates in such a domain-unspecific manner. For instance, BI effects have been reported for tasks that differ in terms of response type—manual, vocal, or foot-pedal responses—[18], indicating that inhibition generalizes to different response modalities. This may suggest the existence of a domain-unspecific inhibitory process that suppresses the representations of the response associated with the interfering task. A second hypothesis is that separate BI mechanisms operate according to the type of input material from which the task representation is generated (e.g., verbal or spatial). Some evidence seems to support this hypothesis. For instance, the finding that the switch cost is larger when switching to the stronger, more dominant between two tasks indicates that the degree of task control is adjusted in a context-sensitive way [19,20].

A classical approach to investigate material-dependency of a cognitive function consists of studying clinical populations characterized by relatively weak and strong areas of performance in definite domains to single out the processing of a specific (for instance, spatial from verbal) material [21]. Therefore, individuals with Williams syndrome (WS) appear to be the ideal participants to examine material specificity of BI. Indeed, WS received a great deal of attention because of the fractionated cognitive profile featuring individuals affected by WS. Specifically, the essential integrity of linguistic abilities has been found since the first observations [22–24] and, with some exceptions [25], confirmed by the following studies [26,27]. Conversely, several aspects of visuo-spatial processing, such as location-encoding and perceptual grouping of elements, orientation discrimination, mental imagery, spatial relationships, and spatial memory, have been consistently found impaired in WS [28–34]. This atypical neuropsychological profile has been related to the atypical brain of individuals with WS. Indeed, the most consistent neuroimaging findings indicate the presence of occipital and parietal cortex abnormalities, which may account for the visuo-spatial deficits occurring in WS [35–38]. Moreover, an atypical cytoarchitecture of the primary auditory cortex [39], a reduced leftward surface temporal planum asymmetry [40], and an increased volume of the superior temporal gyrus have been observed in individuals with WS [37] and associated with their increased sensitivity and attraction to language and to sounds in people with WS [37].

Thus, the characteristic cognitive profile of the individuals with WS, supported by their brain structure, offers the possibility to gain reliable information on the material dependency of inhibitory control. If BI operates on task representations regardless of the type of input from which task representations are generated (e.g., verbal or spatial), one would expect to find BI effects in both typically developing (TD) children and individuals with WS regardless of the type of input material (spatial or verbal); conversely, if BI mechanisms operate according to the type of input material that generates task representations (verbal or visuo-spatial) one would expect BI effects for both verbal and spatial input material in TD individuals, while BI effects should be present for verbal, but not visuo-spatial, input material in individuals with WS.

## Materials and Methods

### Overview of the experiments

Based on the dissociation between the ability to process verbal and visuo-spatial information in individuals with WS, we implemented three task-switching paradigms with verbal or spatial material that was to be processed by a group of individuals with WS and a mental age- and gender-matched group of typically developing (TD) children. Experiment 1 (Verbal Task-Switching) primarily required processing of verbal stimuli, whereas Experiments 2 (Visuo-Spatial Task-Switching) and 3 (Visuo-Spatial Task-Switching in an ecological environment) entailed spatial features of the stimuli (i.e., spatial localization) to be processed in a small computerized or a large-scale radial maze, respectively.

### Participants

Thirteen individuals with WS and 13 TD children were recruited to participate in the study and matched for mental age (ME) and gender. All individuals with WS (7 males and 6 females) and TD children individuals (7 males and 6 females) were right-handed and native Italian speakers, and belonged to upper-middle class families, as assessed by a short questionnaire that was given to the participants’ parents. In participants with WS, the clinical diagnosis of WS was confirmed by the genetic investigation FISH (fluorescent *in situ* hybridization), demonstrating the characteristic deletion on the chromosome band 7q11.23. In addition to a clinical and genetic diagnosis of WS, selection criteria for study recruitment included normal or corrected-to-normal vision. The TD children were recruited from local schools, and their parents reported that they were in good health. Exclusion criteria were reports of neurological signs and history of language delay or learning disabilities.

The participants’ cognitive level was measured using the short version of the Leiter-R intelligence scale [41]. Accordingly to the matching criteria, the mean mental age of the two groups did not differ [*F*(1, 24) = 1.44, *p* = .24, η_P_^2^ = .057; WS: $\bar{x}$ = 6.4 yrs ± .25; TD: $\bar{x}$ = 6.7 yrs ± .21]. Conversely, the two groups differed for mean intellectual quotient (IQ) [*F*(1, 24) = 233.45, *p* < .00001, η_P_^2^ = .91; WS: $\bar{x}$ = 56.4 ± 2.61; TD: $\bar{x}$ = 105.8 ± 1.91] and for chronological age (CA) [*F*(1, 24) = 36.9, *p* < .00001, η_P_^2^ = .61; WS: $\bar{x}$ = 19.9 yrs ± 2.1; TD: $\bar{x}$ = 6.8 yrs ± .2]. Moreover, visuo-motor integration [42] and memory functions [43] were also assessed (Table 1).

**Table 1:** Statistical comparisons of performances of WS and TD participants.

Cognitive domain	WS	TD	Group effect
	Mean ± SEM	Mean ± SEM	*F*(1, 24); *p; η*_*P*_^*2*^
**Visuo-motor integration**	11.46± 0.64	15.31 ± 0.41	25.08; 0.00004; 0.51
**Visuo-spatial short-term memory**	2.08 ± 0.18	3.62 ± 0.21	30.76; 0.00001; 0.56
**Visuo-object short-term memory**	2.54 ± 0.14	2.85± 0.19	1.65; 0.21; 0.064

The study was approved by the Ethics Committee of Children’s Hospital "Bambino Gesù”, Rome, Italy [protocol number: 486LB]. The parents of all participants gave informed written consent and the study was conducted per the Declaration of Helsinki.

### General procedures

The entire experimental procedure lasted three days. On each day, participants performed one of the three task-switching experiments. The order of presentation of the three experiments was counterbalanced among participants.

## Experiment 1: Verbal Task-Switching

This experiment tested the BI effect in a task-switching paradigm that required participants to process verbal stimuli without tapping their spatial components. The paradigm was adapted to the cognitive features of participants with WS—i.e., stimuli presentation time and single trial duration were lengthened, total experiment duration was minimized so as not to burden attentive demands, and words indicating familiar animals were used as stimuli.

### Materials and procedure

**Fig 1:**
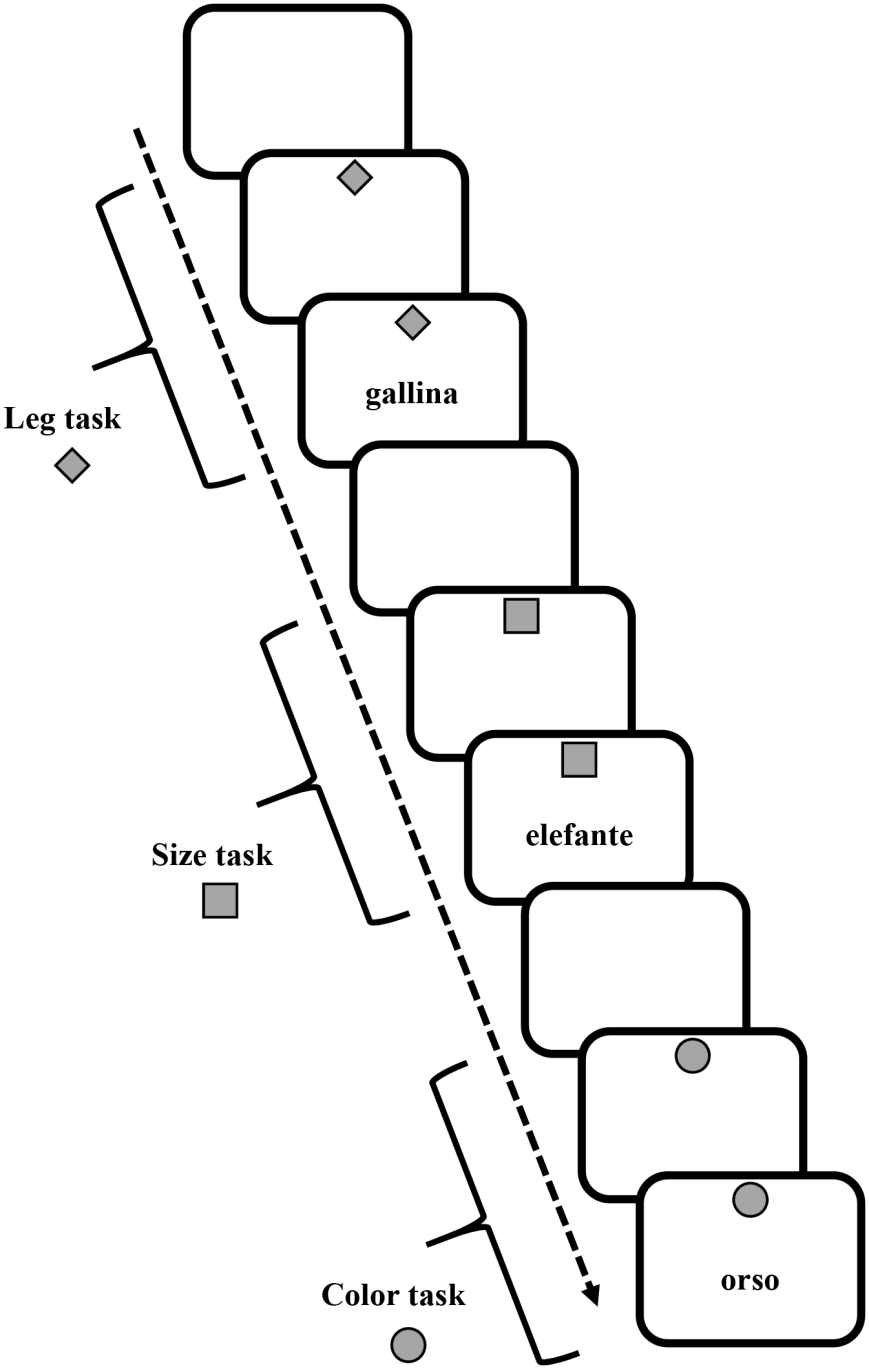
Verbal Task-Switching. Schematic representation of task cues and stimuli in the verbal task-switching paradigm (Experiment 1). (Gallina: chicken; elefante: elephant; orso: bear).

Participants sat in front of a computer screen (distance 60 cm), on which the target and cue stimuli appeared. White or gray (with equal probability) target stimuli (7° width x 4° height visual angle) comprised names of animals (elephant, bear, ostrich, chimpanzee, turtle, rabbit, chick, rooster) that appeared at the center on the screen. At the top of the screen, a gray cue (7° width x 7° height of visual angle) in various shapes (square, diamond, circle) appeared on a white background (Fig 1).

In each trial, participants were asked to perform one of three tasks with the current stimulus, determining: (a) the number of legs of the animal (2-footed or 4-footed); (b) the actual size of the animal (small or large); and (c) the color of the word (white or gray). The upcoming task was pre-cued by the diamond to indicate an upcoming “leg number” task, the square for the “size” task, or the circle for the “color” task. In each trial, the cue appeared for 1000 ms and was followed by the target stimulus. For all tasks, the cue and target stimuli remained on the screen until a response was given or 10,000 ms had elapsed. For all trials, the response was followed by white screen for 700 ms, after which the next cue appeared.

Participants pressed one of two response buttons (the “A” and “L” keys) on a computer keyboard with the left and right index finger, respectively. Participants responded to the 2-footed, small, or white-written words of animals with the left hand and to the 4-footed, large, or gray-written words of animals with the right hand. Each participant underwent 189 trials in which randomized series of non-alternating (CBA), alternating (ABA), and repetition (AAA) three-trial sequences (triplets) appeared. In a non-alternating CBA sequence, three different tasks were executed (legs-color-size, size-color-legs, etc.); in an alternating ABA sequence, the same task was performed for the first and third trial (legs-color-legs, size-color-size, etc.); in a repetition AAA sequence, the same task was performed for three successive trials (color-color-color, size-size-size, etc.). The sequence of tasks was randomly created with the constraint that the number of trials for each of the three tasks (i.e., legs, color and size) had to be counterbalanced. Since in a purely random (without replacement) selection procedure the probability to have three consecutive trials of the same task (AAA) is smaller than the probability to have three consecutive trials of different tasks (CBA) or a N– 2 task repetition (ABA), the number of repetition sequences (AAA) was necessarily smaller that the number of non-alternating (CBA) and alternating (ABA) sequences. Thus, 24 alternating, 27 non-alternating, and 12 repetition sequences were presented. Given the randomized presentation and lack of interval between triplets, participants were unaware that different sequences were presented. The task was made up of three blocks of about 5 min each, with an inter-block interval of about 2 min. Thus, the Experiment 1 lasted about 20 min.

#### Parameters

*Reaction Times* (*RTs*) on the third trials of the alternating sequences (ABA) were compared to those of the non-alternating sequences (CBA) to determine the *BI effect*. *RTs* on the third trials of the repetition sequences (AAA) were compared to those of the non-alternating (CBA) and alternating (ABA) sequences to estimate the *switch cost*. Only triplets for which participants responded correctly to all trials were used to compute the BI and switch cost effects. The *percentage of correct responses* was also computed.

### Results

**Fig 2:**
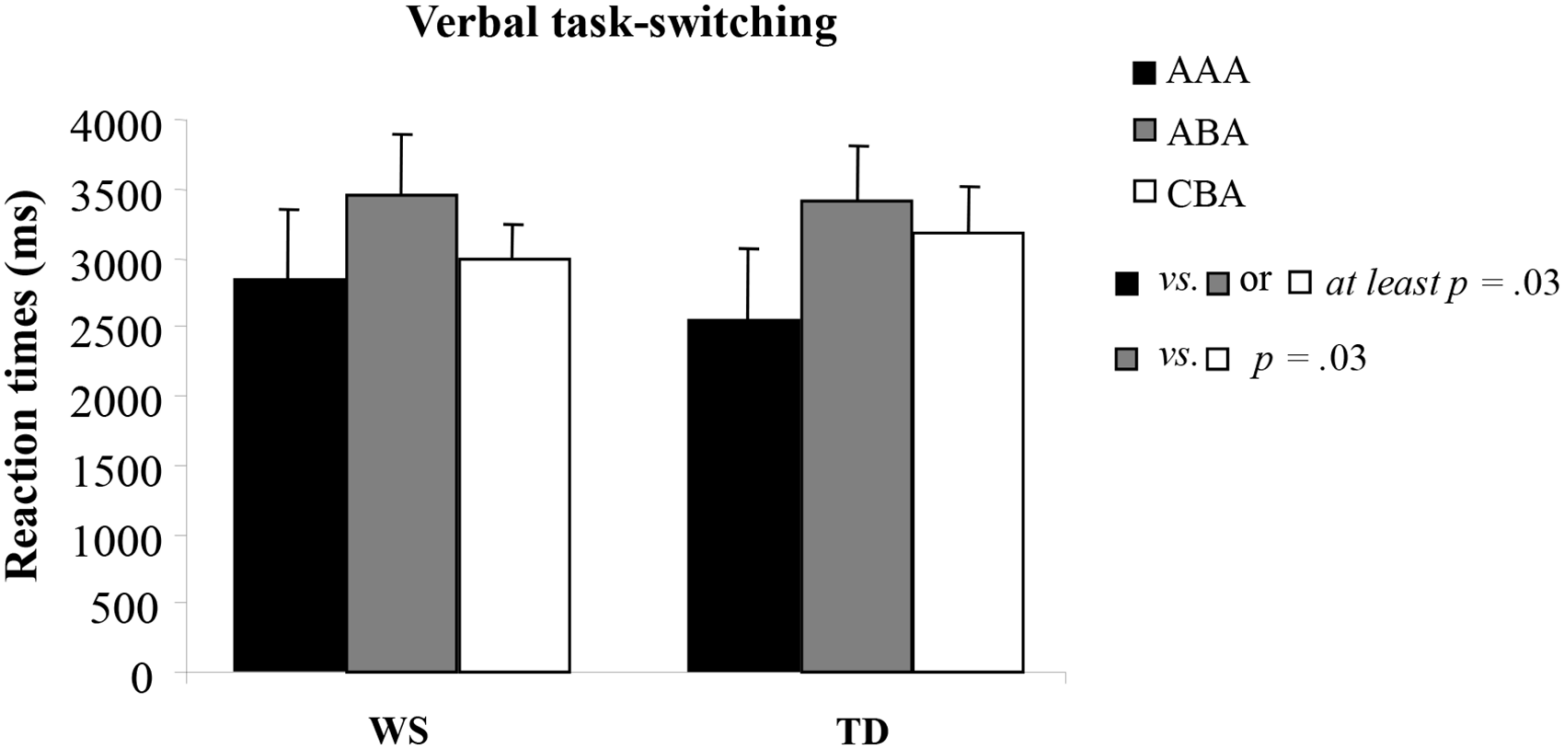
Performances of WS and TD participants in the Verbal Task-Switching. Reaction times (RTs) in repetition (AAA), alternating (ABA) and non-alternating (CBA) sequences of trials for WS and TD participants. Data are expressed as unweighted mean ± SEM.

A one-way ANCOVA (covariates: MA, IQ, CA) on the *percentage of correct responses* of WS and TD groups showed that both groups had a similar level of accuracy [WS: $\bar{x}$ = 69.50 ± 2.15; TD: $\bar{x}$: 91.12 ± 2.08; *F*(1, 21) = 0.40, *p* = 0.53, η_P_^2^ = .02]. To estimate BI and switch cost effects, a two-way ANCOVA (covariates: MA, IQ, CA) was performed on *RTs* through a mixed factorial design with Group (WS and TD groups) and Sequence (repeated measures, AAA, ABA, and CBA sequences) as factors. This analysis revealed a significant sequence effect [*F*(2, 48) = 10.03, *p* = .0002, η_P_^2^ = .29], whereas the group effect [*F*(1, 21) = 1.11, *p* = .30, η_P_^2^ = .05] and the group by sequence interaction [*F*(2, 48) = 1.01, *p* = .37, η_P_^2^ = .04] were not significant. Post hoc comparisons on the significant sequence effect revealed that BI and switch cost effects were present in both groups, as indicated by the slower *RTs* in the ABA versus CBA sequence and slower *RTs* on switch (ABA or CBA) compared to repetition trials (AAA) (Fig 2). In order to test for the reliability of the results, the *RTs* have also been estimated through a bootstrap procedure [44]. Namely, for each participant and experimental condition a set of *RTs* has been created by resampling (with replacement) 200 times from the set of original data. On each resampling, the average *RT* (theta statistic) was computed. The average value of the 200 theta statistic is the bootstrap estimate of the *RT* for that participant in that condition. The same set of analyses described above was replicated on the bootstrap *RTs*. The results were virtually identical to those above reported, thus they will not be discussed any further.

## Experiment 2: Visuo-Spatial Task-Switching

Experiment 1 showed that individuals with WS exhibited a typical BI effect in the verbal task-switching paradigm, suggesting that they exert inhibitory control on verbal information. The aim of the Experiment 2 was to determine whether participants with WS showed a BI effect also when the task switching procedure requires processing of visuo-spatial information. To this aim, we subjected the same participants to a visuo-spatial task-switching paradigm that required them to switch among spatial positions. This paradigm has been shown [9] to be effective in assessing the BI effect in shaping the strategies that individuals use to efficiently explore a changing environment, dismissing previously visited locations.

**Fig 3:**
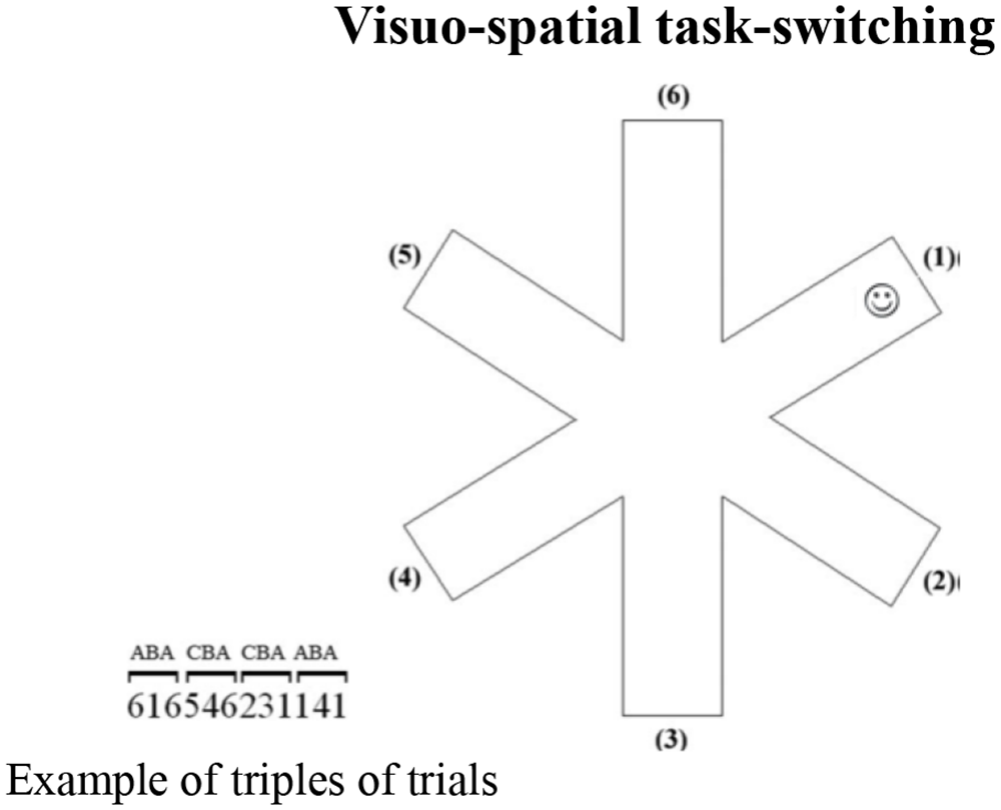
Visuo-Spatial Task-Switching. Schematic representation of the visuo-spatial task-switching paradigm (Experiment 2). On the left, an example of triplets of trials (CBA and ABA). The numbers indicate the arms of the 6-arm star.

Specifically, we asked participants to search for a target (a smiley face) that was hidden at the end of one arm of a 6-arm star on a computer touchscreen (Fig 3).

### Materials and procedure

At the beginning of each trial, the outline of a 6-arm star appeared at the center of a touchscreen on a subtending white background (14.25° width x 14.25° height of visual angle). The target was a small, yellow, stylized representation of a smiley face (smiley) (.48° width x .48° height of visual angle), hidden on each trial at the distal location of one of the six arms (Fig 3). On each trial, participants had to touch the arms successively until they found the smiley. When the smiley was discovered, a new trial began. Trials were organized in triplets in order to have CBA and ABA sequences. Specifically, in the CBA sequences, the smiley was in a different arm, randomly selected, on each trial of the triplet. In the ABA sequences, the smiley was at the same arm, randomly selected, on the first and third trials of the triplet. Notably, participants were unaware of such an organization, as no visual information was given about the kind of sequence each trial belonged to. Each participant performed 180 trials because each sequence was made by 3 trials. Thus, 90 trials were needed to have 30 ABA sequences and other 90 trials to have 30 CBA sequences. The task was made up of three blocks of about 5 min each, with an inter-block interval of about 2 min. Thus, the Experiment 2 lasted about 20 min. On each trial, all the arms touched by the participants were recorded.

#### Parameters

We measured the *number of arms* touched on finding the smiley; the *number of errors*, based on re-exploration of the same arm in the same trial; *perseverations*, defined as exploration of the same arm (e.g., 2–2) or the same string of arms (1-2-1-2) twice consecutively in the same trial; and the *starting arm*, which was the first arm that was touched in each trial. The percentage of response pairs for which the arms were adjacent was also computed as *adjacency values* (e.g., touching arms 2–3 or 6–5 or 6–1 sequentially) (Fig 3). Lower adjacency values reflect more scattered exploration, whereas higher values indicate more systematic and regular exploration [45].

### Results

**Fig 4:**
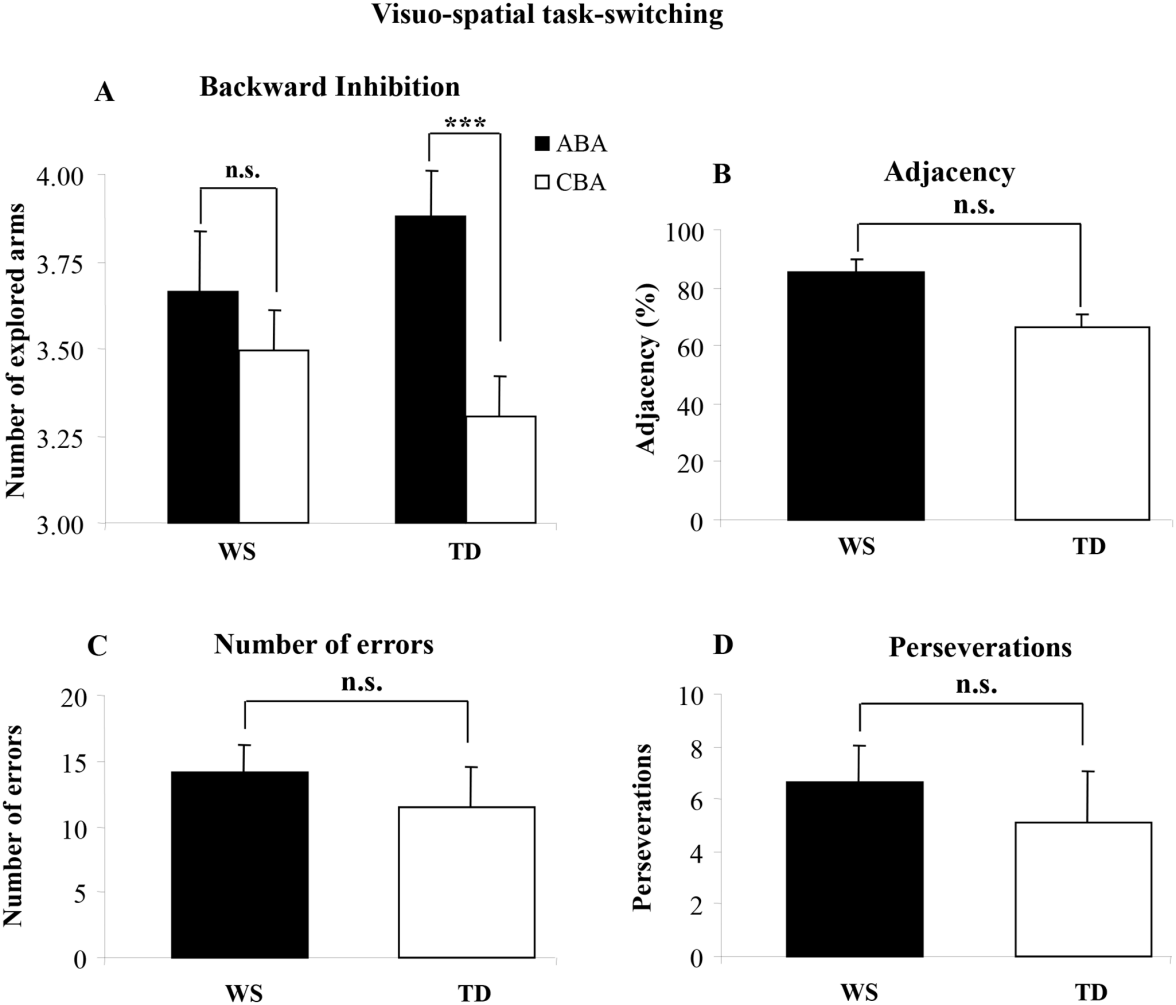
Performances of WS and TD participants in the Visuo-Spatial Task-Switching. **A: Backward inhibition**. Number of explored locations on finding the target in alternating (ABA) and non-alternating (CBA) sequences of trials for WS and TD participants. **B: Adjacency**. The percentage of response pairs for which the arms were adjacent for WS and TD participants. **C: Number of errors**. Number of re-exploration of the same arm in the same trial for WS and TD participants. **D: Perseverations**. Number of exploration of the same arm (e.g., 2–2) or the same string of arms (1-2-1-2) twice consecutively in the same trial for WS and TD participants. Data are expressed as unweighted mean ± SEM. The asterisks indicate the significance level of the post-hoc comparisons between groups (*** *p*< .0001).

A two-way ANCOVA (Group x Sequence; covariates: MA, IQ, CA) on the *number of arms* revealed a not significant group effect [*F*(1, 21) = .003, *p* = .96, η_P_^2^ = .0001], indicating that both groups reached the same level of performance. The sequence effect [*F*(1, 24) = 17.11, *p* = .0004, η_P_^2^ = .42] and the interaction [*F*(1, 24) = 5.11, *p* = .033, η_P_^2^ = .17] were significant. Post-hoc comparisons on interaction revealed that TD participants explored more locations on the ABA than CBA sequences (*p* = .0009), indicating that BI was present in TD children. Conversely, there was no significant difference in the numbers of arms participants with WS explored on the ABA and CBA sequences (*p* = .19) (Fig 4A).

Notably, in the analysis on the *number of errors* (TD $\bar{x}$ = 11.46 ± 3.12; WS $\bar{x}$ = 14.08 ± 2.16), *perseverations* (TD $\bar{x}$ = 5.1 ± 1.98; WS $\bar{x}$ = 6.67 ±1.41), *starting arm* (TD $\bar{x}$ = 28.4 ± 2.19; WS $\bar{x}$ = 45.2 ± 6.40), and *adjacency values* (TD $\bar{x}$ = 66.16% ± 4.77; WS $\bar{x}$ = 85.77% ±4.22), there were no differences between groups [one-way ANCOVAs (covariates: MA, IQ, CA): *number of errors*: *F*(1, 21) = .79, *p* = .38, η_P_^2^ = .04; *perseverations*: *F*(1, 21) = 2.76, *p* = .11, η_P_^2^ = .12; *starting arm*: *F*(1, 21) = .75, *p* = .39, η_P_^2^ = .035; *adjacency value*: *F*(1, 21) = 0.57, *p* = .46, η_P_^2^ = .03] (Fig 4).

In order to test for the reliability of the results, the *number of arms* has also been estimated through a bootstrap procedure [44]. Namely, for each participant and experimental condition a set of arm numbers has been created by resampling (with replacement) 200 times from the set of original data. On each resampling, the average number of arms (theta statistic) was computed. The average value of the 200 theta statistic is the bootstrap estimate of the number of arms for that participant in that condition. The same set of analyses described above was replicated on the bootstrap numbers of arms. The results were virtually identical to those above reported, thus they will not be discussed any further.

## Experiment 3: Visuo-Spatial Task-Switching in an Ecological Environment

Experiment 2 showed that TD participants explored more locations before finding the target in the ABA versus CBA sequences compared to participants with WS, who explored the same number of locations in the ABA and CBA sequences. This finding suggests that only in TD participants the target location became inhibited and hence was less likely to be explored in the successive trial (the BI effect). However, because the target location changed in most of the trials in Experiment 2, the larger number of explored arms in the ABA than in the CBA conditions could be accounted for by the experimental design favoring random exploration. To determine the function of the BI in modulating the spontaneous spatial exploration and to control for the above-mentioned confounding factor in the design of Experiment 2, we performed a further experiment using a large-scale radial maze [9] to analyze the spontaneous navigational abilities. To avoid favoring random exploration of the environment by design, the target was fixed between trials, allowing us to determine which locations were visited on each trial, instead of the overall number of visited locations. If BI favors the exploration of new sites, then participants should visit the previous locations less frequently than new sites, generating more CBA than ABA responses, despite the target location remaining constant.

### Materials and procedure

#### Apparatus

**Fig 5:**
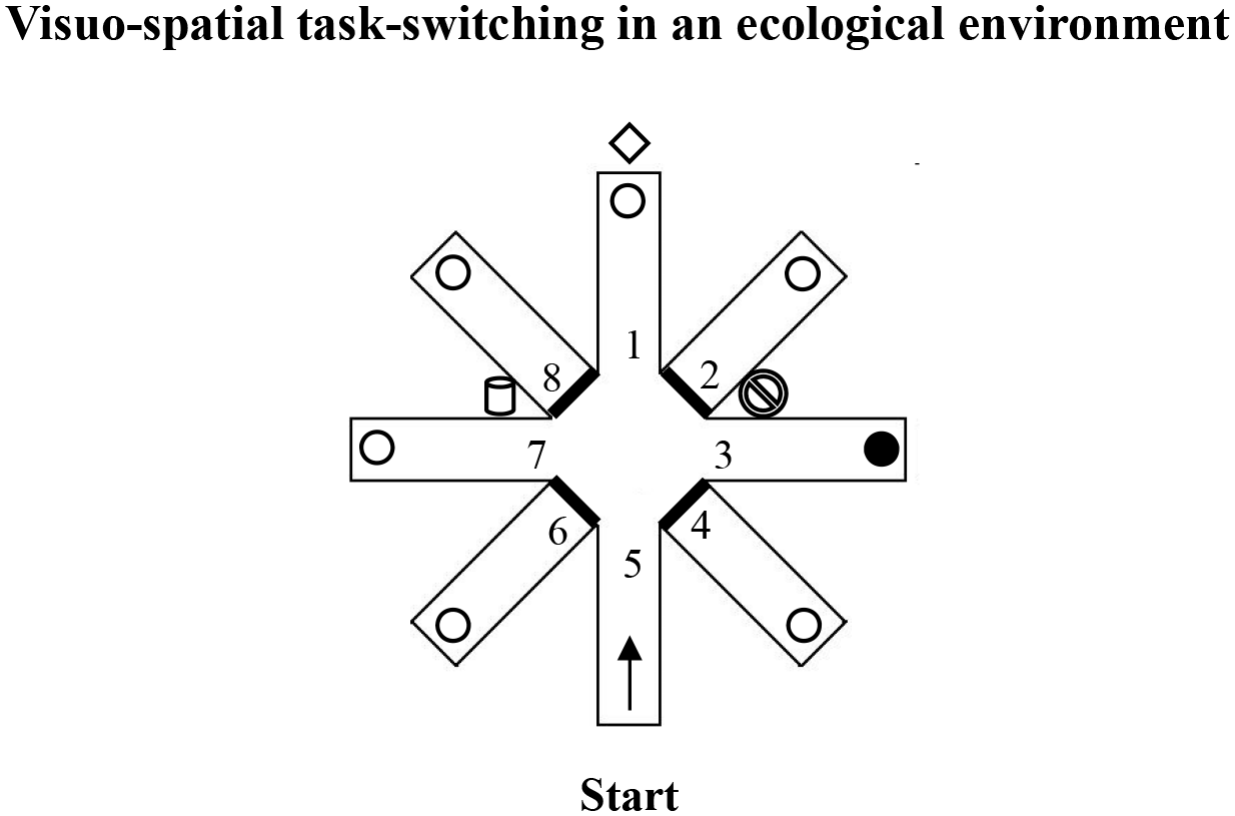
Visuo-Spatial Task-Switching in an ecological environment. Schematic representation of the visuo-spatial task-switching paradigm in an ecological environment (Experiment 3).

The large-scale radial arm maze (RAM) comprised a round central platform (3 m in diameter) with 8 arms (80 cm wide x 11 m long), radiating like the spokes of a wheel. White and red ribbons formed a barrier that marked the sides of each arm and forced the participants to return to the center of the starting platform before entering another arm, thus preventing them from “cutting corners.” At the end of each arm lay an orange plastic bucket (18 cm wide x 28 cm high) that contained a reward (a plastic coin). The 8 arms were virtually numbered as in Fig 5. The RAM was placed outdoors in a large square and was surrounded by extra-maze cues (e.g., trees, buildings, pavement, streetlamps) that were fixed throughout the experiment. Attention was paid to control the intra- and extra-maze environment with regard to cues, the location of the buckets, and the position of the experimenter. Participants could see and access the maze only during the experimental sessions.

The proximal ends of arms 2, 4, 6, and 8 were closed. The participant started from the distal end of arm 5 and had to search for a reward unpredictably put always in the same arm 3 until she/he made 3 consecutive visits in the rewarded arm (the criterion) (Fig 5). After visiting an arm, regardless of discovering the reward, the participant was guided out of the maze and hidden from the maze for 30 s before starting another trial. During this interval, the bucket at the end of arm 3 was always reinforced. The duration of Experiment 3 varied from about 2 to 18 min, according to the number of arms explored to reach the criterion by the single participants.

#### Parameters

We recorded the *number of trials to criterion* to determine performance level and the *number of ABA and CBA* sequences to evaluate BI.

### Results

**Fig 6:**
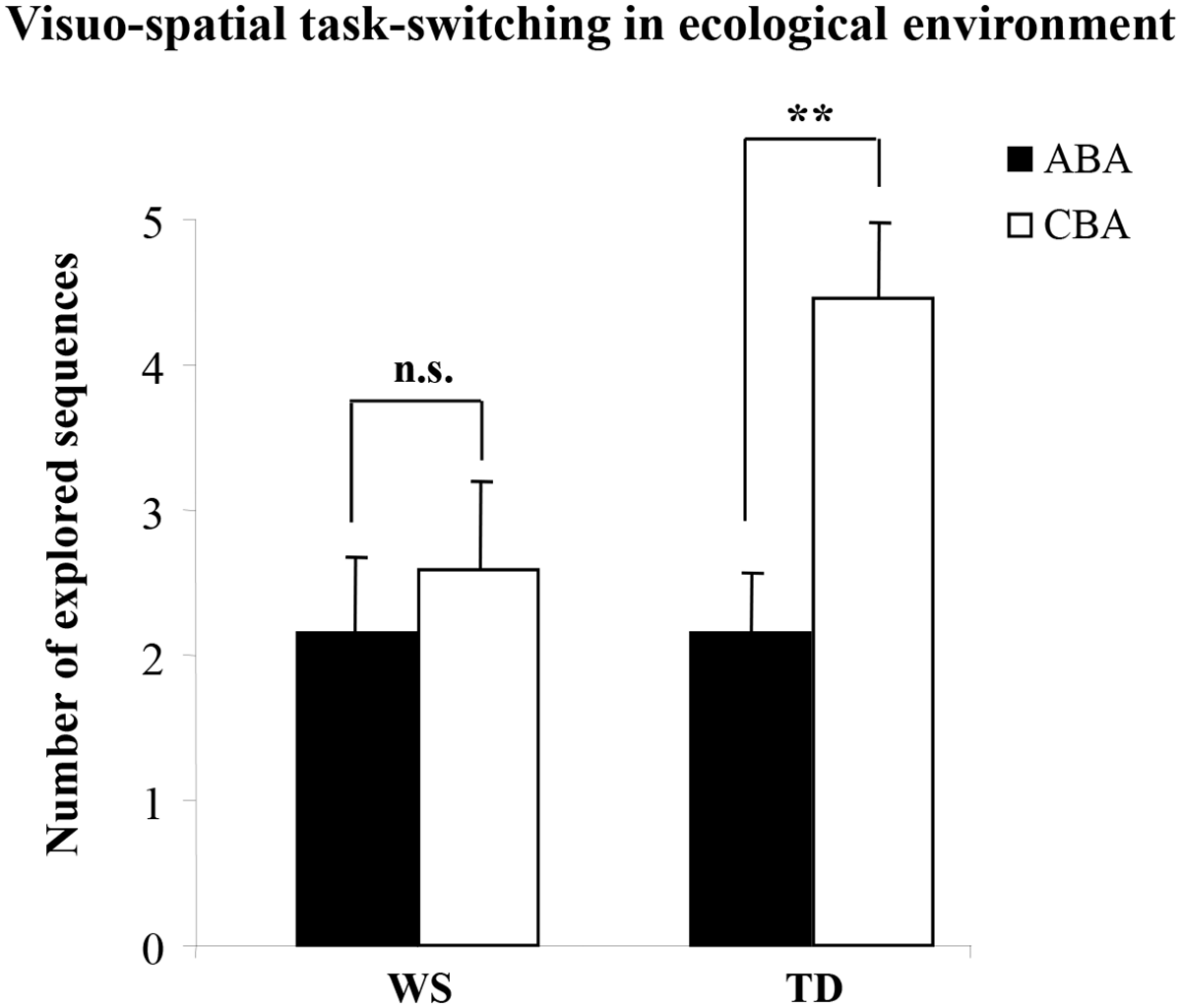
Performances of WS and TD participants in the Visuo-Spatial Task-Switching in an ecological environment. Number of sequences of locations (ABA and CBA) that the participants spontaneously explored. Data are expressed as unweighted mean ± SEM. The asterisks indicate the significance level of the post-hoc comparisons between groups (** *p*< .005).

The *number of trials to criterion* was similar between the WS and TD groups (WS $\bar{x}$ = 10.84 ± 1.58; TD $\bar{x}$ = 10.92 ± 1.36), indicating that they had the same level of performance [one-way ANCOVA (covariates: MA, IQ, CA): *F*(1, 21) = .16, *p* = .70, η_P_^2^ = .007]. A two-way ANCOVA (Group x Sequence; covariates: MA, IQ, CA) on the *number of CBA and ABA sequences* revealed a not significant group effect [*F*(1, 21) = 1.61, *p* = .22, η_P_^2^ = .07], whereas the sequence effect [*F*(1, 24) = 10.49, *p* = .004, η_P_^2^ = .30] and interaction [*F*(1, 24) = 4.94, *p* = .036, η_P_^2^ = .17] were significant. Post-hoc comparisons on interaction revealed that the TD participants generated more CBA than ABA sequences (*p* = .002). Conversely, participants with WS generated almost the same number of CBA and ABA sequences (*p* = .75) (Fig 6). These results demonstrate that only in TD the pattern of exploration was driven primarily by the inhibition of the previously explored location (i.e., BI effect) and not by the target location being varied (Experiment 2) or fixed (Experiment 3). Results also confirm that participants with WS do not show visuo-spatial BI effects.

## Discussion

A domain-general account for control processes (monitoring, inhibition, selection, error detection, etc.) is frequently assumed to exist [46], although there is evidence that favors domain-specificity [47]. However, no study has yet examined whether the inhibitory mechanism underlying the BI effect is an amodal process or whether material-specific effects do exist. The study of BI in individuals with WS offered a unique opportunity to clarify the issue of the material dependency of inhibitory control due to the well-known dissociation between verbal (preserved) and visuo-spatial (compromised) abilities characterizing the neuropsychological profile of individuals with WS.

To this aim, we compared the BI effect of individuals with WS to that of mental age- and gender-matched TD children in three experiments. Experiment 1 (verbal task-switching) was based on the processing of verbal stimuli, whereas Experiments 2 (visuo-spatial task-switching) and 3 (visuo-spatial task-switching in an ecological environment) entailed the processing of the spatial location of stimuli in a small-scale computerized maze and a large-scale radial maze, respectively. It is important to consider that the effects of the BI on task performance can be assessed in different ways. Previous studies have mostly focused on speeded response times [1] in the attempt to evaluate how inhibition affects the time necessary to the cognitive system for a rapid adaptation to changing task demands. In these cases, the BI is assessed by means of reaction times differences, as it is the case of the present Experiment 1. However, inhibitory control can affect performance not only by reducing the response readiness of the system but also by modulating the activation level of task representations in working memory [48], thus affecting the availability of specific representations for future task operations (e.g., in a spatial search strategy) [9]. Assessing the BI effects across speeded (e.g., Experiment 1) and non-speeded tasks (Experiments 2 and 3) allows a deeper investigation of the different ways in which inhibitory control can affect our behaviour. However, direct comparisons of BI effects between speeded and non-speeded paradigms are not possible. Accordingly, in the present study the BI effects were only compared between different groups (WS *vs*. TD) and never the BI effects were directly compared between different experiments.

The main result of Experiment 1 was the significant increase of RTs when both WS and TD participants switched to a task set that had been abandoned two trials earlier (ABA sequence). This effect is consistent with the presence of an inhibitory process that alters the cognitive configurations that are to be abandoned and, most importantly, it indicates that BI is present in individuals with WS when verbal information must be processed (Fig 2A).

A stark contrast in results was observed using task-switching paradigms based on visuo-spatial processing. In Experiment 2, TD children adapted their search strategy to the previous outcomes, as indicated by the greater number of explored locations in the ABA than in the CBA sequences. Conversely, participants with WS explored the same number of locations in both the ABA and CBA sequences, indicating that their search strategy was independent of where the target was found on the previous trials (Fig 4A). It might be hypothesized that this result was due to the weak visuo-spatial short-term memory of individuals with WS (Table 1) [21].

However, the number of errors and perseverations (re-exploration of the same arm in the same trial) revealed no significant differences between groups. Since these parameters represents a reliable index of visuo-spatial short-term memory (given you re-explore the same arm on the same trial only whether you do not keep track of which arm you had already explored before), the results of participants with WS cannot be accounted for by their weak visuo-spatial short-term memory.

The lack of a spatial BI effect in individuals with WS evidenced in Experiment 2 was confirmed by the results of Experiment 3, in which the spontaneous generation of ABA and CBA responses was examined in an ecological environment. TD children chose the arm to be explored based on their previous choices. They generated fewer ABA than CBA responses, a clear indication that they tended not to return to previously explored (hence, inhibited) arms. Conversely, participants with WS generated a similar number of ABA and CBA responses, indicating that their responses were independent of the previously explored locations (Fig 6). As in Experiment 2, no difference between groups in overall performance was observed, as evidenced by the similar number of trials to criterion. This confirms that the lack of BI effect in individuals with WS was not due to impairment in visuo-spatial information processing. The not significantly different number of ABA sequences in the two groups supports this interpretation. Indeed, poor short-term memory would have produced a high number of ABA sequences, since not remembering which arm has been already explored makes it more likely to be explored again.

To sum up, the BI deficit in the participants with WS was present when the paradigm facilitated both random (i.e., when the target location changed from trial to trial in Experiment 2), and ordered (i.e., when the target location was fixed across trials in Experiment 3) exploration. Conversely, the pattern of exploration by TD participants indicates the presence of a patent BI effect in both Experiment 2 and Experiment 3.

Conclusively, it is noteworthy that the two groups of participants showed the same levels of accuracy in both the verbal- and spatial-based task switching paradigms, and the individuals with WS failed to show a BI effect in the spatial-based, but not in the verbal-based, task switching paradigm. Hence, the present study points toward the existence of a domain-specificity of the BI. However, we are aware that this interpretation has to be taken with some caution. Individuals with WS have a genetic neurodevelopmental disorder that impacts their frontal lobes and diffuse white matter connectivity, in addition to their right hemisphere. Thus, in this population other aspects of executive functioning (working memory, attention, problem solving, organization, planning, perseveration, etc.) mediated by these networks are impaired, as previously demonstrated by some of us [49,50]. However, the faceted results of individuals with WS here found (impaired visuo-spatial BI and preserved verbal BI) support the indication that they were not an epiphenomenon of a general executive functions impairment. In any case, it will be useful to replicate the present study in populations affected by neurodevelopmental disorders without intellectual disability. Furthermore, future studies on populations with more pure and focal insult of right hemisphere will help making conclusions sounder and not confounded by pervasive executive functioning weaknesses. In this regard, some clues come from a study by Mayr and colleagues [51] in which the BI effect was assessed in patients with focal lesions in the left or in the right prefrontal cortex. While patients with left frontal insult showed a preserved BI, patients with right frontal insult did not show any sign of BI. Furthermore, an fMRI study by Dreher & Berman (2002) [52] showed that the ABA vs. CBA contrast is associated with increased activation in the right frontal cortex.

### Domain-general and domain-specific accounts of executive functions

Control processes that select and coordinate goal-related information are strictly associated with top-down activity from the prefrontal lobes. Specifically, these processes are believed to enhance the representations that underlie the current behavior and inhibit irrelevant or inappropriate information. Top-down control is necessary when automatic responses are not purposeful, as in novel or difficult situations, or when a conflict among separate representations arises, as when old representations interfere with new ones.

Whether top-down control is domain-general or domain-specific remains controversial. For instance, a domain-general account of attentional control is supported by findings that a single common network of fronto-parietal regions is activated by attentional transitions and switches between task sets, regardless of the perceptual information on which they are based [53,54], although other studies have provided evidence of domain-specific networks that mediate attentional control [55]. Similarly, it is debated whether the working memory affects selective attention in a domain-general or domain-specific way. The domain-general account is supported by the load in one content domain (phonological) influencing processing in another content domain (visuo-spatial) [56,57]. Alternatively, the domain-specific account is supported by effects being observed only when memory maintenance and attentional processing operate in the same domain [13,58,59]. Even studies on resolution between competing representations have generated contrasting results [12,60].

Notably, it has not yet been addressed at all whether the BI is domain-general or domain-specific. Most of the studies examining the mechanisms of BI had implicitly accepted the domain-general account. For instance, studies on executive function deficits in clinical populations have used a single task-switching paradigm [61–63]. Further, in healthy subjects studies aimed to clarify some characteristics of BI, such as the function of cues [64–67], the effects of cue-stimulus interval [15,65,68,69], and the effects of variables, such as mindfulness and stress [70], have assumed BI to be domain-general. However, our results rather indicate a domain-specific account of the BI. They are nicely coherent with the findings showing that voluntary task control is a multi-faceted process, in which attentional, intentional and strategic components [71], as well as different types of inhibitory mechanisms, such as the competitor rules suppression [72], contribute to the performance. They are also coherent with the notion that multiple neural mechanisms deal with specific conflicts, at the perceptual-, motor-, emotional-, representational-level [73–75]. Among others, the BI has been proposed as a conflict-solving process aimed at reducing the proactive interference from old to new representations. However, when and how the BI is triggered is still an open issue. Schuch and Koch (2003) [17] proposed a response-selection triggering mechanism of the BI. Specifically, the residual shift costs would reflect the persisting inhibition of a previous task-set as a consequence of the conflict in response selection arising when the same motor response (e.g., pressing the A key on the keyboard) is associated with different tasks (e.g., 2-legged, small, white animal names). Several models in the task-switching literature are compatible with this response-selection account of BI [5,76,77], though some also distinguish different components of task sets, including a stimulus set and a response set [78]. Each of these components could potentially trigger the BI depending on the stage of processing wherein the conflict between the tasks is detected. Accordingly, some evidence have been provided that task inhibition can occur at many different levels of task processing [67], depending on whether the conflict between different tasks arises at the perceptual [16,64,79,80] or at the response level [18,81].

Interestingly, control processes have been defined as ‘reactive’ and “prospective’ [12], the former being conflict-triggered (e.g., when the tasks share the same stimuli) and the latter being related to the advanced task preparation (e.g., task predictability). Particularly, Egner (2008) [12] proposed that the conflict-triggered adjustment of processing priorities is domain-specific. Our results indicate that the same domain-specific perspective may also apply to the BI, though the characteristics of BI do not allow it to be classified as solely “reactive” or “prospective” [12,15,65,82–84]. This issue should be further examined in future research to determine whether cognitive control processes are domain-specific independently of their “reactive” or “prospective” nature.

### BI and working memory

The BI is assumed to operate on task representations that are active in working memory during task execution, and that are supposed to interfere with the execution of the correct task. The BI would reduce the activation level of the preceding, most competing task, modulating the relative activation differences between appropriate and inappropriate task representations in working memory. Owing to the central role that BI plays in modulating the activation level of task representations in working memory, a future challenge for theories of cognitive control would be to incorporate BI functioning within modern theories of working memory.

Recently, the relation among task switching, working memory and executive control has begun to be more deeply investigated [85]. Using a task span procedure wherein the number of tasks a person could remember and execute perfectly (task span) and the ability to switch from one task to another are assessed in the same experimental design, Logan (2004) [85] found that the task span is unaffected by the amount of task switching required, indicating that storage in working memory does not trade off with the executive processing required for task switching. Both Logan’s (2004) [85] and present findings are more consistent with the theories that assume multiple executive control systems than with the theories that assume a single executive control system. Thus, a theoretical challenge for future research is to formulate models of executive control that integrate the functioning of modality-specific BI, task switching and working memory in a coherent view.

### BI and wayfinding

Overall, the findings of the present study prompt us in advancing a speculative, but intriguing, link between BI process and wayfinding. In everyday navigation, we do not rely on any single navigational process, and instead we constantly switch among different navigational strategies to utilize cues that may be inconsistently available, or to operate on small and large scales. These strategies can be classified by the reference frame, given some of them operate in relation to the body's changing position and orientation (egocentric strategy), and others in relation to a fixed external coordinate system (allocentric strategy) [86]. For example, allocentric processing relies on using distal environmental cues to find a novel route, while egocentric processing depends on encoding a sequence of body movements that allows following a known route. Allocentric and egocentric strategies are associated with hippocampus and caudate nucleus, respectively [87,88,89,90] that provide input to the prefrontal cortex. It has been advanced that just the switching between strategies is fundamentally important to wayfinding [91,92]. It may be suggested that inhibitory control in visuo-spatial domain, as assessed by BI, shapes the strategies that individuals use to efficiently explore ever-changing environments by dismissing previously visited locations [9]. This inhibitory process reduces the interference from previously stored information, and facilitates the instantiation of new information, allowing individuals to adapt flexibly to continuously changing environments.

Furthermore, to successfully navigate in an environment it is necessary to activate general and schematic representation of the environment (cognitive map), “zooming” on the sector in which the individual is actually moving. While the general representation is kept stable, the zoom is shifted to update it according to individual’s translations. BI might support this process by reducing the activation level of the previous representation in the spatial working memory buffer [8]. A crucial requisite of memory systems is the maintenance in working memory of the active representation of stored information, avoiding at the same time it may interfere with the formation of new representations. An important role in this process might be played by BI, which does not allow previous representations to interfere with the next ones.
